# Phylogenetic and expression analysis of the *NPR1*-like gene family from *Persea americana* (Mill.)

**DOI:** 10.3389/fpls.2015.00300

**Published:** 2015-04-29

**Authors:** Robert Backer, Waheed Mahomed, Bianca J. Reeksting, Juanita Engelbrecht, Enrique Ibarra-Laclette, Noëlani van den Berg

**Affiliations:** ^1^Forestry and Agricultural Biotechnology Institute, University of PretoriaPretoria, South Africa; ^2^Department of Genetics, Fruit Tree Biotechnology Program, University of PretoriaPretoria, South Africa; ^3^Laboratorio Nacional de Genómica para la Biodiversidad-Langebio/Unidad de Genómica Avanzada, Centro de Investigación y Estudios Avanzados del – Instituto Politécnico NacionalIrapuato, México; ^4^Red de Estudios Moleculares Avanzados, Instituto de Ecología A.C.,Xalapa, México

**Keywords:** avocado, *Phytophthora cinnamomi*, NPR1, expression analysis, salicylic acid, jasmonic acid, *pathogenesis*-*related*

## Abstract

The NONEXPRESSOR OF PATHOGENESIS-RELATED GENES1 (NPR1) forms an integral part of the salicylic acid (SA) pathway in plants and is involved in cross-talk between the SA and jasmonic acid/ethylene (JA/ET) pathways. Therefore, NPR1 is essential to the effective response of plants to pathogens. Avocado (*Persea americana*) is a commercially important crop worldwide. Significant losses in production result from Phytophthora root rot, caused by the hemibiotroph, *Phytophthora cinnamomi*. This oomycete infects the feeder roots of avocado trees leading to an overall decline in health and eventual death. The interaction between avocado and *P. cinnamomi* is poorly understood and as such limited control strategies exist. Thus uncovering the role of NPR1 in avocado could provide novel insights into the avocado – *P. cinnamomi* interaction. A total of five *NPR1*-like sequences were identified. These sequences were annotated using FGENESH and a maximum-likelihood tree was constructed using 34 NPR1-like protein sequences from other plant species. The conserved protein domains and functional motifs of these sequences were predicted. Reverse transcription quantitative PCR was used to analyze the expression of the five *NPR1*-like sequences in the roots of avocado after treatment with salicylic and jasmonic acid, *P. cinnamomi* infection, across different tissues and in *P. cinnamomi* infected tolerant and susceptible rootstocks. Of the five *NPR1*-like sequences three have strong support for a defensive role while two are most likely involved in development. Significant differences in the expression profiles of these five *NPR1*-like genes were observed, assisting in functional classification. Understanding the interaction of avocado and *P. cinnamomi* is essential to developing new control strategies. This work enables further classification of these genes by means of functional annotation and is a crucial step in understanding the role of NPR1 during *P. cinnamomi* infection.

## Introduction

Plants recognize and react to external threats much like any other living organism, eliciting a response to combat disease (Robert-Seilaniantz et al., 2011). Defense responses against biotrophic and hemibiotrophic pathogens are mainly dependent on the salicylic acid (SA) pathway (Glazebrook, 2005). Plants challenged by a biotrophic pathogen show a substantial increase in endogenous SA, a subsequent hypersensitive response (HR) at the site of infection and the onset of systemic acquired resistance (SAR; Malamy et al., 1990; Metraux et al., 1991; Rasmussen et al., 1991; Gaffney et al., 1993; Delaney et al., 1994). SAR is an important part of plant defense, providing long term, broad spectrum resistance which is effective against a wide variety of fungal, viral and bacterial pathogens at tissues distal to the initial site of infection (Frederich et al., 1996; Sticher et al., 1997; An and Mou, 2011; Robert-Seilaniantz et al., 2011). Increases in SA concentration have been conclusively linked to the establishment of SAR, for instance, exogenous application of SA or one of its biologically active analogs, 2,6-dichloroisonicotinic acid (INA) and benzo (1,2,3) thiadiazole-7-carbothioic acid *S*-methyl ester (BTH), is able to induce SAR (White, 1979; Ward et al., 1991; Lawton et al., 1995). Conversely, plants that express the transgene *nahG* which encodes for a salicylate hydroxylase, lack functionally active SA and are SAR compromised (Gaffney et al., 1993; Bi et al., 1995; Friedrich et al., 1995; Lawton et al., 1995).

The quest to discover the SA receptor led to the discovery of NONEXPRESSOR OF PATHOGENESIS-RELATED GENES1 (NPR1), a transcription co-factor protein encoded for by *NPR1* (Cao et al., 1994). The majority of described NPR1 proteins contain ankyrin repeat and Broad Complex, Tramtrack and Bric a brac/Pox virus and Zinc finger (BTB/POZ) domains (Cao et al., 1997; Hepworth et al., 2005; Spoel et al., 2009). These domains are essential for protein–protein interactions and enable NPR1 to function as a co-activator (Cao et al., 1997; Rochon et al., 2006). In *Arabidopsis* NPR1 is found as an oligomer within the cytoplasm of uninduced cells and changes in SA concentration lead to an altered redox environment within the cell, supporting the nuclear localization of NPR1 in its monomeric form (Mou et al., 2003). It is worth noting that NPR1 is constitutively localized within the nucleus of several plant species, yet the perception of a SA signal is still required for the expression of *pathogenesis-related* (*PR*) genes (Kinkema et al., 2000; Le Henanff et al., 2009; Maier et al., 2011).

Multiple NPR1-like proteins seem to be present in most, if not all, plant species. Phylogenetic analysis of this family suggests the existence of three functionally distinct clades (Hepworth et al., 2005; Zhang et al., 2006; Peraza-Echeverria et al., 2012). Members of the first clade, AtNPR1 and AtNPR2, are mostly associated with positive SAR regulation (Cao et al., 1997, 1998). The second clade, AtNPR3, and AtNPR4, is associated with negative SAR regulation, yet is required for effective SAR induction (Liu et al., 2005; Zhang et al., 2006). The third clade, AtBOP1, and AtBOP2, is associated with the development of lateral organs (Hepworth et al., 2005). Phylogenetic analysis has since included NPR1-like proteins from multiple plant species (Le Henanff et al., 2009; Peraza-Echeverria et al., 2012; Shao et al., 2013), and although phylogenetic analysis alone is insufficient for functional annotation it may provide a basis for understanding functional variation (Liu et al., 2005; Zhang et al., 2006).

The most extensively studied member of the *Arabidopsis* NPR1-like family is AtNPR1. Mutants of this protein are more susceptible to virulent pathogens and display compromised expression of *PR* genes when compared to plants expressing wild-type *NPR1* (Glazebrook et al., 1996; Cao et al., 1997). Complementation of these *npr1* mutants using wild-type *NPR1* restores the expression of *PR* genes as well as pathogen resistance and the induction of SAR (Cao et al., 1997). Various plants overexpressing *NPR1* show increased *PR* gene expression and pathogen resistance (Cao et al., 1998; Friedrich et al., 2001; Malnoy et al., 2007; Le Henanff et al., 2011; Chen et al., 2012; Kumar et al., 2012). Overexpressing *OsNPR1*, the ortholog of *AtNPR1* in rice, results in an increased resistance to bacterial blight, yet these transgenic plants show an increased susceptibility to herbivores (Yuan et al., 2007). Interestingly, herbivore hypersensitivity is alleviated when NPR1 is constitutively localized to the nucleus (Yuan et al., 2007). These results suggest that NPR1 is involved in the antagonistic cross-talk between the SA and jasmonic acid/ethylene (JA/ET) pathways, a theory supported by several other studies (Spoel et al., 2003; El Oirdi et al., 2011). Thus, NPR1 is considered the master regulator of defense responses in plants.

Additionally, NPR1 interacts with several members of the TGACG motif-binding factor (TGA) family of basic leucine zipper protein (bZIP) transcriptions factors (Zhang et al., 1999; Despres et al., 2000; Zhou et al., 2000). These transcription factors associate with the *as-1-like* (TGACG) promoter element within *PR* gene promoters and are responsible, at least in part, for their expression (Fan and Dong, 2002; Zhang et al., 2003). Moreover, the DNA binding affinity of TGA factors is increased when associated with NPR1 (Despres et al., 2000; Fan and Dong, 2002) and NPR1 may also deactivate the repression of *PR* genes by certain TGA factors (Rochon et al., 2006). This interaction describes the basis of NPR1-dependant gene expression, yet the realistically more complex mechanism involves several other factors (Li et al., 1999; Weigel et al., 2001; Chern et al., 2005; Zhang et al., 2006; Zwicker et al., 2007).

Evidence suggests that NPR3 and NPR4 are essential to establishing SAR even though they suppress NPR1-dependant gene expression (Liu et al., 2005; Zhang et al., 2006; Fu et al., 2012). Both NPR3 and NPR4 act as adaptors for Cullin 3 (CUL3) E3 ligase-facilitated ubiquitinylation and subsequent proteasome degradation of NPR1 (Fu et al., 2012). The degradation of NPR1 serves dual roles; turn-over of NPR1 as well as suppression of NPR1-dependant gene expression in SA naïve cells and cells undergoing HR (Spoel et al., 2009). The expression of NPR1-dependant genes during non-stress conditions and HR increases fitness costs and prevents the establishment of HR, respectively (Rate and Greenberg, 2001; Heidel et al., 2004). In SA naïve cells NPR4 strongly interacts with NPR1 thus preventing increases in expression of *PR* genes (Fu et al., 2012). Moreover, increased SA concentrations interrupt this interaction and increase the affinity of NPR3 for NPR1 (Fu et al., 2012). At the site of HR where SA concentrations are the highest NPR1 is rapidly degraded, while at distal tissues with intermediate SA concentrations NPR3 merely facilitates sufficient turn-over of NPR1 (Fu et al., 2012). The turn-over of transcription factors ensures optimal expression of target genes (Salghetti et al., 2000; Collins and Tansey, 2006), as seen for NPR1. Thus by responding to the concentration of SA, NPR3, and NPR4 prevent the untimely expression of *PR* genes, fine-tuning the defense response. It is therefore clear that understanding the role of the NPR1-like family is an important part of understanding defense responses in plants.

Avocado is an economically important fruit crop with an annual worldwide gross production value of US $ 3835 million^1^. The fruit are highly nutritious and contain high levels of monounsaturated fats making them popular for use in a wide variety of culinary products. The greatest threat to production is Phytophthora root rot (PRR), caused by the hemibiotrophic oomycete pathogen *Phytophthora cinnamomi* Rands (Hardham, 2005). Infection by *P. cinnamomi* results in decreased water and nutrient absorption due to necrosis of the avocado feeder roots, leading to a decline in tree health and eventual death ensuing economic losses (Zentmyer, 1984; Coffey, 1987). With a wide host range of more than 3000 plant species and the ability to persist in the environment (Weste, 1983; Hardham, 2005), effective control of *P. cinnamomi* is limited.

The use of phosphite trunk injections, tolerant rootstocks (e.g., Dusa^®^) and organic mulching practices are currently utilized by industry as methods for controlling PRR (Coffey, 1987; Giblin et al., 2005). Phosphite trunk injections have been a dependable method for over 30 years (Darvas et al., 1984; Pegg et al., 1985; Coffey, 1987; Kaiser et al., 1997; Giblin et al., 2005), yet evidence suggests that *P. cinnamomi* has the potential to develop decreased sensitivity against this fungicide (Duvenhage, 1994; Dobrowolski et al., 2008). Similar observations occur for metalaxyl, another decidedly effective fungicide (Darvas et al., 1984). Moreover, the lengthy selection process for PRR tolerant rootstocks (Gabor and Coffey, 1991; Menge, 1999; Kremer-Köhne and Mukhumo, 2003) limits the introduction of novel tolerant rootstocks, possibly providing the pathogen with an opportunity to overcome host tolerance.

Although biochemical and histological studies have provided some insight into the avocado – *P. cinnamomi* interaction (Phillips et al., 1987; Botha and Kotze, 1989; Bekker et al., 2006; Sánchez-Pérez et al., 2009; García-Pineda et al., 2010), research on the molecular characteristics of this interaction have only recently gained attention (Mahomed and van den Berg, 2011; Reeksting et al., 2014). Our current understanding of the incompatible *Arabidopsis thaliana* – *P. cinnamomi* interaction provides limited information on compatible interactions. For example, in *Arabidopsis* the JA/ET pathway seems to be essential to *P. cinnamomi* resistance (Rookes et al., 2008), yet in avocado SA inhibits growth and colonization (García-Pineda et al., 2010). Such conspicuous differences between non-host and host interactions highlight the need to elucidate the host specific interaction between avocado and *P. cinnamomi* on a molecular level.

Thus defining the role of NPR1 in avocado could potentially provide novel insights into the avocado – *P. cinnamomi* interaction. This is the first study aimed at discovering and characterizing *NPR1*-like genes in *Persea americana*. We have discovered five *NPR1*-like genes from *P. americana* which harbor the ankyrin repeat and BTB/POZ domains and show sequence similarity to other known *NPR1*-like genes. Phylogenetic analysis reveals that the predicted protein sequences of these genes can be resolved into the three known phylogenetic clades of the NPR1-like family. We describe the expression of these genes in Dusa^®^, a PRR tolerant avocado rootstock, across five time points during treatment with SA, MeJA, and *P. cinnamomi* using RT–qPCR. Additionally, we measured the basal expression levels for each transcript across six different tissues. The findings of this study provide an invaluable resource for further study and functional characterization of the NPR1-like family in avocado. Future efforts could focus on intracellular interactions and localization as well as overexpression of defense related *PaNPR1*-like genes in wild-type and *npr1* mutant *Arabidopsis*.

## Materials and Methods

### Sequence Annotation and Phylogenetic Analysis

Five *NPR1*-like gene sequences were obtained from the *P. americana* genome (Unpublished data). Sequences were arbitrarily assigned identifiers as follows: *PaNPR1*, *PaNPR2*, *PaNPR3*, *PaNPR4,* and *PaNPR5*. Open reading frames (ORF’s) were annotated using the online prediction tool FGENESH with the *Vitis vinifera* genome-specific parameters selected (Solovyev et al., 2006). Exon/intron positions of predicted CDSs were visualized using GSDS software v2.0 (Guo et al., 2007). Percentage amino acid similarity was calculated using SIAS^2^. Protein domains were predicted using PROSITE (Sigrist et al., 2010). Sequences were submitted to GenBank^3^: *PaNPR1* [GenBank: KR056089], *PaNPR2* [GenBank: KR056090], *PaNPR3* [GenBank: KR056091], *PaNPR4* [GenBank: KR056092], and *PaNPR5* [GenBank: KR056093].

### Phylogenetic Analysis

Additional NPR1-like protein sequences from other plant species were attained online at NCBI^4^ in order to perform alignments (**Table 1**). Sequences were aligned using Clustal W software v2.1 (Thompson et al., 1994). The best substitution model for the alignment was determined and subsequently used in construction of a maximum likelihood (ML) phylogenetic tree using the tools available in MEGA software v5.2 (Tamura et al., 2011). The initial tree was constructed using the neighbor-joining (NJ) method (Saitou and Nei, 1987) and bootstrapping (1000 replicates) was used to determine confidence.

**Table 1:** Additional NPR1-like protein sequences from other plant species.

Species	Identifier	Accession number
*Arabidopsis thaliana*	AtNPR1	[GenBank: NP_176610]
*A. thaliana*	AtNPR2	[GenBank: NP_194342]
*A. thaliana*	AtNPR3	[GenBank: NP_199324]
*A. thaliana*	AtNPR4	[GenBank: NP_193701]
*A. thaliana*	AtBOP1	[GenBank: NP_001190116]
*A. thaliana*	AtBOP2	[GenBank: NP_181668]
*Populus deltoides*	PdNPR1-1	[GenBank: AEY99652]
*P. deltoides*	PdNPR2	[GenBank: AEE81755]
*Beta vulgaris*	BvNPR1	[GenBank: AAT57640]
*Hordeum vulgare subsp. vulgare*	HvNPR1	[GenBank: CAJ19095]
*Sorghum bicolor*	SbNPR1	[GenBank: XP_002455011]
*Helianthus annuus*	HaNPR1	[GenBank: AAT57642]
*Glycine max*	GmNPR1-1	[GenBank: ACJ45013]
*G. max*	GmNPR1-2	[GenBank: ACJ45015]
*Physcomitrella patens*	PhNPR-like1	[GenBank: XP_001757508]
*P. patens*	PhNPR-like2	[GenBank: XP_001759240]
*Vitis vinifera*	VvNPR1.1	[GenBank: XP_002281475]
*V. vinifera*	VvNPR1.2	[GenBank: XP_003633057]
*Oryza sativa*	OsNPR1	[GenBank: AAX18700]
*O. sativa*	OsNPR2	[GenBank: ABE11616]
*O. sativa*	OsNPR3	[GenBank: ABE11618]
*O. sativa*	OsNPR5	[GenBank: ABE11622]
*Gossypium hirsutum*	GhNPR1	[GenBank: ABC54558]
*Ipomoea batatas*	IbNPR1	[GenBank: ABM64782]
*Solanum lycopersicum*	LeNPR1	[GenBank: AAT57637]
*Nicotiana tabacum*	NtNPR1	[GenBank: AAM62410]
*Capsicum annum*	CaNPR1	[GenBank: ABG38308]
*Musa spp. AAA*	MNNPR1A	[GenBank: ABI93182]
*Musa spp. AAA*	MNNPR1B	[GenBank: ABL63913]
*Musa spp. ABB*	MdNPR1	[GenBank: ACJ04030]
*Malus x domestica*	MpNPR1-1	[GenBank: ACC77697]
*Pyrus pyrifolia*	PpNPR1-1	[GenBank: ABK62792]
*Populus trichocarpa*	PtNPR1.1	[GenBank: XP_002308281]
*P. trichocarpa*	PtNPR1-like	[GenBank: XP_002323261]

*Accession numbers for several NPR1-like protein sequences. Sequences were used to construct a maximum likelihood (ML) phylogenetic tree along with the five predicted NPR1-like protein sequences from Persea americana*.

### Plant Material

One-year-old clonal PRR-tolerant Dusa^®^ rootstock plantlets were provided by Westfalia Technological Services (Tzaneen, South Africa). Two phytohormone treatment groups were assigned and treated with 70 ml sodium salicylate (NaSA) solution (5 mM NaSA (Sigma–Aldrich, St. Louis, MO, USA), 0.1% Tween^®^ 20 (v/v) (Sigma–Aldrich, St. Louis, MO, USA) or 70 ml methyl jasmonate (MeJA) solution (5 mM MeJA (Sigma–Aldrich, St. Louis, MO, USA), 0.1% ethanol (99.9%), 0.1% Tween^®^ 20 (v/v)). A third treatment group was inoculated with 20 ml *P. cinnamomi* zoospore suspension (3 × 10^5^ spores/ml) and 70 ml *P. cinnamomi* mycelial suspension. Each treatment contained three biological replicates with two plants per replicate. Control plants were either treated with 70 ml NaSA control solution (0.1% Tween^®^) or 70 ml MeJA control solution [0.1% Tween^®^, 0.1% ethanol (99.9%)]. Each control group contained three biological replicates with one plant per replicate. Plants were randomly assigned to either the treatment or control groups. All treatments and controls were applied directly to the soil at the base of the plant. Roots were harvested for all treatment and control groups at 6, 12, 18, 24, and 96 h. Samples were snap frozen in liquid nitrogen and stored at -80∘C. Biological replicates were homogenized using the IKA^®^ Tube Mill control (IKA^®^, Staufen, Germany) until a fine consistency was attained.

Mature grafted trees located at Westfalia (Tzaneen, South Africa) were used for the collection of tissue samples. Six tissue types were selected: feeder roots, mature green stems, mature green leaves, unripe fruit as well as stems and leaves from flush growth (young material). Samples were taken from a single orchard block which contained clonal Hass fruitstocks grafted onto clonal PRR-tolerant Duke 7 rootstocks. Fifteen trees were randomly selected from which two samples of each tissue were taken for each individual tree. Samples were snap frozen in liquid nitrogen and stored at -80∘C. Tissue samples were randomly allocated to three groups of five trees, individual tissue samples from each group were then pooled and homogenized using the IKA^®^ Tube Mill control (IKA^®^).

### *Phytophthora cinnamomi* Infection

Zoospores were produced by placing *P. cinnamomi* colonized blocks of V8 agar (20% V8 juice (v/v), 0.25% CaCO_3_, agar 17g.l^-1^) into 90 mm Petri dishes containing 2% V8 broth for 3 days to allow sufficient mycelial growth. Cultures were then rinsed three times with dH_2_O and run-off stored for use as mycelial suspension. Filtered stream water was then added and cultures left under UV light for 2–3 days until sufficient sporangia formation was observed. Cultures were then cold-shocked at 4∘C for 45 min and placed at room temperature for 1 h to allow zoospore release. Sufficient zoospore release and motility was monitored via microscopy. Inoculation was carried out immediately by pouring both the zoospore and mycelial suspension directly onto the soil at the base of the plants.

### Nested PCR

Total genomic DNA was isolated from inoculated root samples. Nested LPV3 PCR was then performed in order to confirm successful infection of plant roots by *P. cinnamomi* as described by Engelbrecht et al. (2013). Results were visualized on 2% TAE agarose gel under non-denaturing conditions.

### RNA Extraction

Total RNA was extracted from homogenized plant material using a modified version of the CTAB extraction method described by Chang et al. (1993). The chloroform: isoamyl alcohol step was repeated four to six times until the volume of the interphase diminished and the supernatant was clear. Samples were resuspended in diethylpyrocarbonate (DEPC) treated water containing 30 U/ml RiboLock RNase Inhibitor (Thermo Fisher Scientific Inc., Leicestershire, UK). RNA concentration and purity was assessed using the NanoDrop^®^ ND-1000 spectrophotometer (Nanodrop Technologies Inc., Montchanin, DE, USA). RNA integrity was assessed on 2% TAE agarose gel under non-denaturing conditions.

Total RNA from *P. cinnamomi* infected tolerant (Dusa^®^) and susceptible (R0.12) avocado rootstocks at 0 h (uninfected control), 6, 12, and 24 h were obtained from Engelbrecht et al. (2013). RNA concentration and purity was assessed using the NanoDrop^®^ ND-1000 spectrophotometer (Nanodrop Technologies, Inc., Montchanin, DE, USA). RNA integrity was assessed on 2% TAE agarose gel under non-denaturing conditions.

### cDNA Synthesis

RNA was purified of any contaminants using the RNeasy MinElute Cleanup Kit (Qiagen Inc., Hilden, Germany) according to the manufacturer’s instructions followed by an on-column RNase-free DNase I (Thermo Fischer Scientific) treatment. cDNA was synthesized using the ImProm-II^TM^ single strand cDNA synthesis kit (Promega Corporation, Madison, WI, USA) according to the manufacturer’s guidelines. First strand synthesis was primed using 0.5 μg random hexamers (Thermo Fisher Scientific). cDNA concentration and purity was assessed using the NanoDrop^®^ ND-1000 spectrophotometer (Nanodrop Technologies). Genomic DNA (gDNA) contamination was assessed using the intron-spanning flavone-3-hydroxylase (F3H) primers as described in (Reeksting et al., 2014).

### RT-qPCR

Primers for reverse transcription quantitative PCR were designed using CLC Genomics Workbench v5.1 (CLC Bio, Qiagen^®^ Inc., Hilden, Germany) and quality assessed on NetPrimer (Premier Biosoft International, Palo Alto, CA, USA). Primers with annealing temperatures between 55 and 60∘C, expected amplicons lengths of <150 bp and quality scores >95.0 (NetPrimer) were synthesized (Inqaba Biotec, Pretoria, South Africa; **Table 2**). Primer specificity was confirmed by performing conventional PCR and sequencing (African Center for Gene Technologies, Pretoria, South Africa) and by the presence of a single melting curve. A 1:3 serial dilution was derived from a comprehensive mix of treated and control cDNA samples. Calibration curves were then performed for each candidate and reference gene across multiple temperatures to ensure that efficiency (E) and correlation (*R*^2^) values were in accordance with MIQE guidelines (Bustin et al., 2009; **Table 3**). All reactions were performed using SensiMix^TM^ SYBR^®^ No-ROX kit (Bioline USA, Inc., Taunton, MA, USA) according to manufacturer’s instructions with a final reaction volume of 10 μl. Thermal cycling was performed on the Bio-Rad^®^ CFX 96 instrument (Bio Rad laboratories., Hercules, CA, USA). Thermal cycling conditions were: 10 min at 95∘C followed by 40 cycles of 10 s at 95∘C, 15 s at T_a_ (**Table 3**) and 10 s at 72∘C, a melting dissociation curve was constructed from 60 to 95∘C at 0.5∘C increments following the final cycle. Three endogenous control genes were used in order to normalize the data for each gene, namely *Actin*, *18S*, and *alpha-1 tubulin* as reported by Reeksting et al. (2014). The stability of the reference genes were analyzed using Bio-Rad^®^ CFX Manager software v1.5 (Bio Rad laboratories; **Table 3**). Normalized relative quantities (fold change) for genes were calculated using the method described by Pfaﬄ (2001). Expression values for each time-point in a specific treatment were calibrated against a control of the corresponding time-point. Cleanup of RT–qPCR products was performed using Zymoclean^TM^ Gel DNA recovery kit (Zymo Research Corporation, Irvin, CA, USA) in preparation for sequencing. Sequencing reactions were performed using BigDye^®^ Terminator v3.1 Cycle Sequencing Kit (Life Technologies, Thermo Fisher Scientific) according to the manufacturer’s guidelines. Reactions were precipitated using 3 M sodium acetate (pH 5.2) and submitted for sequencing (African Center for Gene Technologies, Pretoria, South Africa).

**Table 2:** Primer sequences used in reverse transcription quantitative PCR analysis.

Gene	Forward 5′–3′	Reverse 5′–3′	Expected product length (bp)
*PaNPR1*	TGGCTTATCAGTGCTTGCTC	CCTCCTTATCCTCGTTGTATGC	119
*PaNPR2*	GAACCACTACTAGGAGAAG	TTGCCAGACTAACTCTAC	97
*PaNPR3*	CTTCCCGACTTATTCTACCTTGAG	CGATCTGCTGTACTCCTTGTC	126
*PaNPR4*	AGGTGCTGCTGCTGCTAC	TGGATTCGTGGCTTCTCTATGC	94
*PaNPR5*	GTCGAACAGTTGGCATTG	GAGCACTTTCATCACATCTTC	84
*PaPR1*	GCGGCTGGAAAGGTTTGT	GGGGCTGTAGTTGCAAGT	102

*Primer sequences were designed to amplify fragments no larger than 150bp for each of the five NPR1-like genes identified in P. americana in order to perform RT-qPCR analysis*.

**Table 3:** Reverse transcription quantitative PCR optimization.

Gene	*T*	*E*	*R*	LDR	M1	M2
*18S*	58C	108.2	0.995	1:9–1:729	0.0185	0.0579
*-1 tubulin*	58C	91.8	0.997	1:3–1:729	0.0064	0.2076
*Actin*	58C	101.7	0.995	1:9–1:729	0.0671	0.0512
*PaNPR1*	56C	95.6	0.996	1:3–1:729		
*PaNPR2*	59C	93.2	0.991	1:3–1:729		
*PaNPR3*	63C	96.0	0.992	1:3–1:729		
*PaNPR4*	63C	96.9	0.996	1:3–1:729		
*PaNPR5*	62.5C	93.1	0.991	1:3–1:729		
*PaPR1*	58C	97.6	0.992	1:3–1:729		

*Primer sets of the five NPR1-like genes and PR1 from P. americana as well as endogenous control genes were optimized for annealing temperatures (T) that yielded sufficient efficiency (E) and coefficient of determination (R) values. Linear dynamic range (LDR) indicates the minimum and maximum dilutions used to create a calibration curve. The stability (M-value) of the reference genes is also indicated for SA, MeJA, and Phytophthora cinnamomi treated (M1) and different tissue samples (M2)*.

### Statistical Analysis

A student’s *t*-test was performed to determine significance for quantitative gene expression analysis. SA, MeJA, and *P. cinnamomi* treated samples were compared to respective controls at each time point. Statistical analysis was performed using GraphPad Prism software v6.0.5 (GraphPad Software, Inc., La Jolla, CA, USA). Significance was assessed using a 95% confidence interval. Statistical analysis for various tissue samples was done using JMP 11 (SAS Institute Inc., Cary, NC, USA). Initial analysis was performed using one-way ANOVA followed by Tukey’s HSD test, adhoc. Significance was assessed using a 95% confidence interval. A Mann–Whitney unpaired *t*-test was performed to determine significance for quantitative gene expression analysis. Expression in tolerant plants was compared to that of susceptible plants at each respective time point. Statistical analysis was performed with the Statistics Online Computational Resource package^5^ Significance was assessed using a 95% confidence interval.

## Results

### *In Silico* Identification and Analysis

**FIGURE 1:**
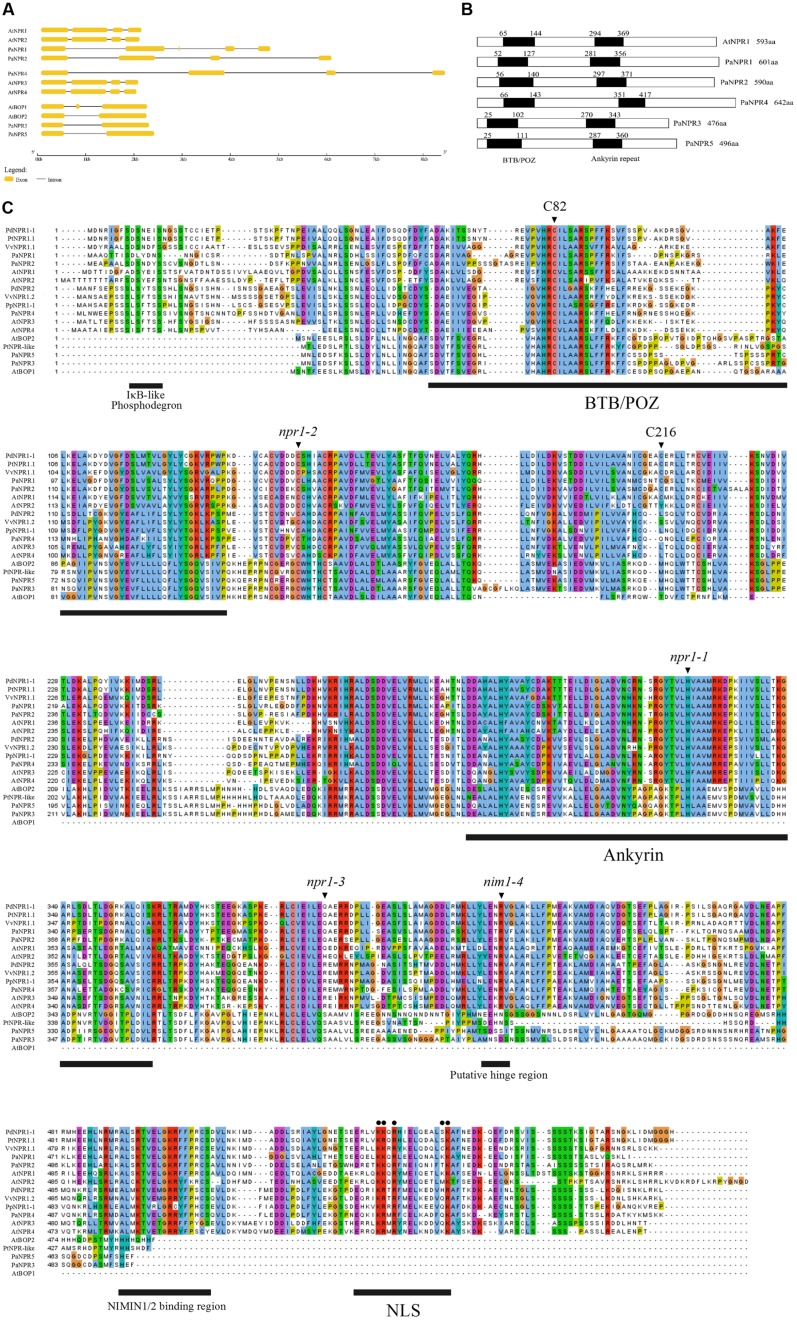
**Exon/intron boundary and predicted coding sequence comparison of *PaNPR1*-like genes with other known *NPR1*-like sequences. (A)** The predicted exon/intron structure of the *NPR1*-like family from *Arabidopsis thaliana* and *Persea americana*. Exons are denoted by yellow boxes while introns are represented by thin black lines. **(B)** A comparison of the positions of the BTB/POZ and ankyrin repeat domains between the PaNPR1-like and AtNPR1-like family of proteins. **(C)** A multiple alignment of PaNPR1-like proteins and several other known NPR1-like proteins from woody plants and *Arabidopsis*. The positions of amino acid changes causing the npr1-1(H), npr1-2 (C), npr1-3 (^∗^), and nim1-4 (R) mutants as well as the positions of the highly conserved cysteine residues at position 82 and 216 in *Arabidopsis* are indicated by black triangles above the alignment. The BTB/POZ and ankyrin repeat domains are indicated by black bars below the alignment. Several important motifs such as the IκB phosphodegron, LENRV hinge region, NIMIN1/2 binding site, and NLS1, are also indicated by black bars. The positions of important amino acids in the NLS1 of AtNPR1 are indicated by black dots above the alignment.

A total of five *NPR1*-like sequences were obtained from the unpublished *P. americana* genome. *PaNPR1*, *PaNPR2*, *PaNPR3*, *PaNPR4,* and *PaNPR5*, code for putative proteins of 601 aa, 590 aa, 476 aa, 642 aa, and 496 aa, respectively. Amino acid analysis revealed that PaNPR1 and PaNPR2 are most similar to AtNPR1 (52.46% identity, 64.08% similarity, and 55.94% identity, 68.35% similarity, respectively) while PaNPR4 is most similar to AtNPR3 (56.74% identity, 66.08% similarity). Furthermore, PaNPR3 and PaNPR5 are highly similar to AtBOP2 (78.90% identity, 83.71% similarity, and 81.57% and identity, 85.18% similarity, respectively). The predicted exon/intron structure further illustrates similarities between the avocado and *Arabidopsis NPR1*-like gene families (**Figure 1A**).

Analysis using PROSITE (Sigrist et al., 2010) reveals that all five PaNPR1-like proteins contain the BTB/POZ and ankyrin repeat domains at similar positions to AtNPR1 (**Figure 1B**). Conversely, only PaNPR1, PaNPR2, and PaNPR4 contain an NPR1-like C-terminal region which has been shown to be an essential component of NPR1 (Cao et al., 1997). The C-terminus contains the nuclear localization signal (NLS), a conserved penta-amino acid motif (LENRV) and a NIM INTERACTING (NIMIN) 1/2 protein binding site (Kinkema et al., 2000; Maier et al., 2011). PaNPR1 contains all five of the conserved basic amino acids that constitute the NLS1, PaNPR2 contains four of the five and PaNPR4 contains several conservative amino acid substitutions (**Figure 1C**). Similarly, the NIMIN1/2 binding region is completely conserved in PaNPR1 with one and three substitutions in PaNPR2 and PaNPR4, respectively, (**Figure 1C**). Furthermore, PaNPR4 contains the putative hinge region (LENRV motif) while PaNPR1 and PaNPR2, respectively, contain a conservative threonine and serine substitution at the third position of this motif (**Figure 1C**). The N-terminal of PaNPR2 contains an IκB-like phosphodegron motif (DSxxxS) which has been shown to be necessary for proteasome-mediated turnover of NPR1 (Spoel et al., 2009) while PaNPR1 contains a similar motif with a serine to lysine substitution at the second position (**Figure 1C**).

**FIGURE 2:**
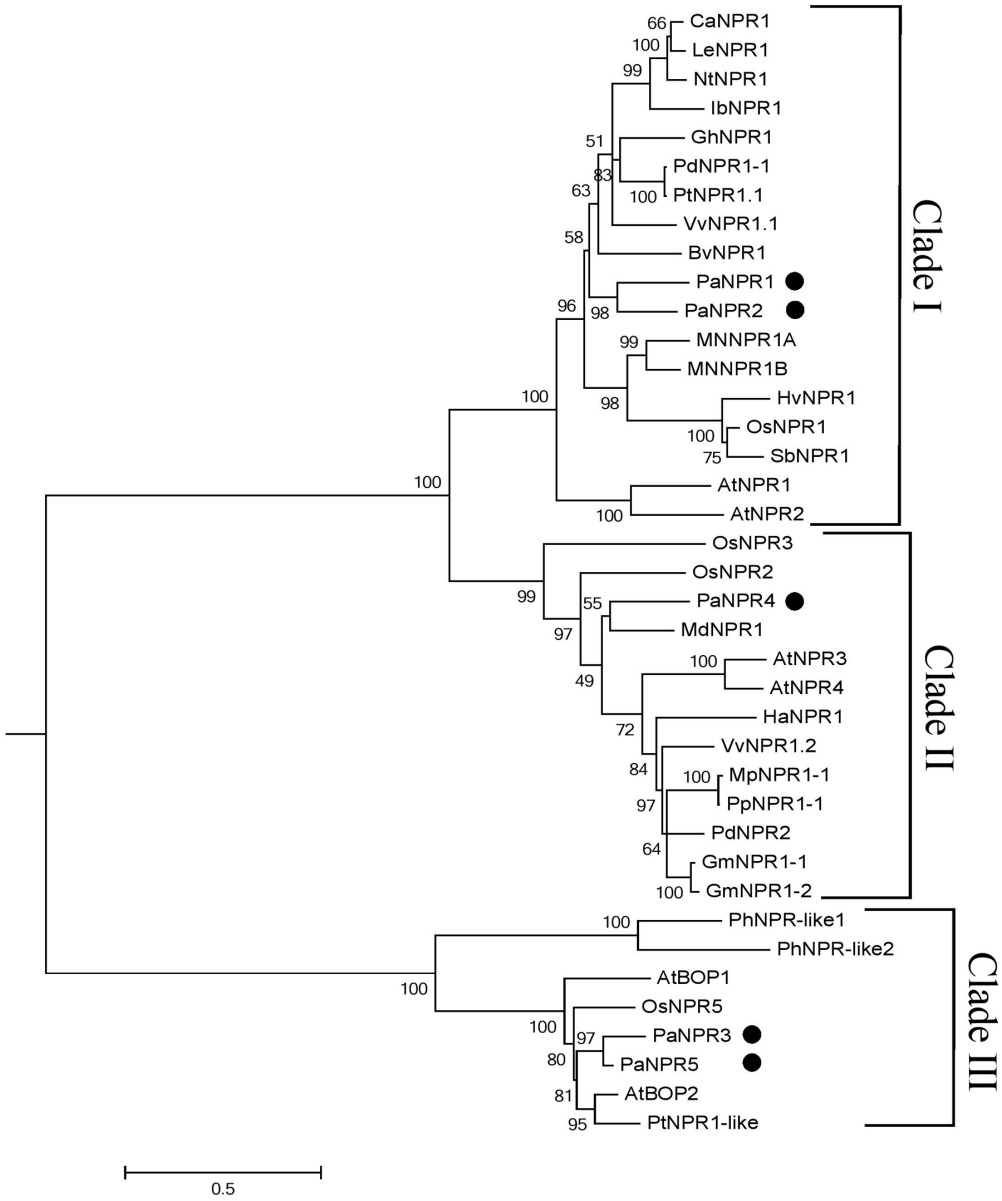
**Phylogenetic analysis of the NPR1-like family of proteins from *P. americana* and several other species.** A phylogenetic tree of five NPR1-like proteins from *P. americana* as well as NPR1-like proteins from other vascular and non-vascular plant species, including the NPR1-like family from *Arabidopsis*. The tree was generated in MEGA software v5.2 using the maximum likelihood (ML) method. A total of 1000 bootstrap replicates were performed and values are indicated above the branch points. The species of origin, identifiers and accession numbers are summarized in **Table 1**.

The PaNPR1-like protein sequences obtained in this study were subjected to phylogenetic analysis together with 34 full-length NPR1-like protein sequences from vascular and non-vascular plant species (**Figure 2**). This analysis reveals that PaNPR1 and PaNPR2 form a distinct group which is closely related to VvNPR1.1 from grapevine (70.83 and 76.33% similarity, respectively) as well as NPR1 from poplar (PtNPR1.1 and PdNPR1-1) and beet (BvNPR1), clustering within the clade containing AtNPR1 and AtNPR2 (**Figure 2**). On the other hand, PaNPR4 clusters within the clade containing AtNPR3 and AtNPR4 (**Figure 2**). Finally, PaNPR3 and PaNPR5 form a distinct group and are closely related to AtBOP2 from *Arabidopsis* (76.02 and 77.90% similarity, respectively; **Figure 2**).

### *PaNPR1*-like and *PaPR1* Response to SA, MeJA, and *P. cinnamomi*

In order to evaluate all five *PaNPR1*-like genes, it is important to gage their expression in response to hormone treatment and pathogen challenge. It is well-known that SA application increases expression of *AtNPR1* approximately twofold within 24 h, similar results are also obtained when *Arabidopsis* is inoculated with *Hyaloperonospora parasitica* (Ryals et al., 1997). Similarly, MeJA application has been shown to result in increased expression of *NPR1* in rice and banana, although to a lesser extent than treatment with SA (Yuan et al., 2007; Endah et al., 2008). Thus, in order to investigate the response of all five *PaNPR1*-like genes, 1 year-old clonal PRR-tolerant Dusa^®^ rootstock plantlets were treated with either SA, MeJA, or inoculated with *P. cinnamomi* and harvested at 6, 12, 18, 24, and 96 h. Furthermore, an ortholog of *Arabidopsis PR1* from *P. americana*, *PaPR1*, was used as a SAR marker (Reeksting et al., 2014).

**FIGURE 3:**
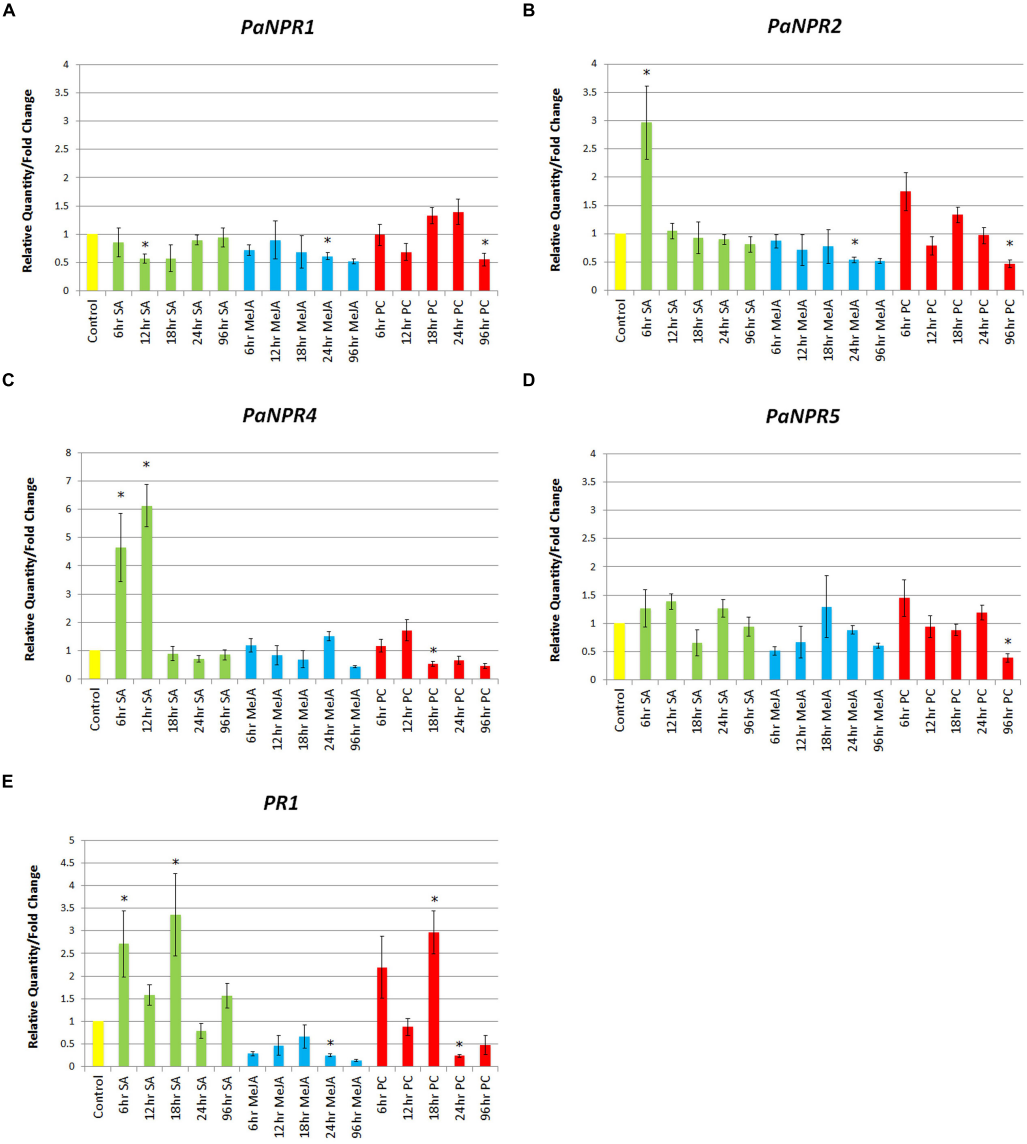
**Expression of *PaNPR1*-like genes as well as *PaPR1* in response to SA, JA, and *Phytophthora cinnamomi* treatment**. Normalized relative quantities (fold change) for **(A)** *PaNPR1*, **(B)** *PaNPR2*, **(C)** *PaNPR4*, **(D)** *PaNPR5*, and **(E)** *PaPR1* were calculated using the method described by Pfaﬄ (2001). The response is indicated by vertical bars across five time points for SA (green), MeJA (blue), and *P. cinnamomi* (red) treated samples, and is labeled on the horizontal axis. The samples were compared to untreated samples harvested at each time point, a representative bar for the controls (yellow) is also indicated. SE for each bar is shown. Significant differences (P < 0.05) between control and treated samples is denoted by an asterisk (^∗^) above the bar.

The expression of *PaNPR1* was significantly down-regulated during SA treatment at 12 h (0.56-fold), returning to basal levels at 96 h (**Figure 3A**). Treatment with MeJA also decreased the expression of *PaNPR1* but at a later time point, 24 h (0.61-fold), yet expression remained low at 96 h (**Figure 3A**). Infection with *P. cinnamomi* decreased the expression of *PaNPR1* at 12 h (0.68-fold) followed by an increase at later time points, similar to treatment with SA, yet differences between treated and control samples were not significant (**Figure 3A**). However, significant down-regulation was seen at 96 h *P. cinnamomi* infection (0.55-fold), similar to treatment with MeJA (**Figure 3A**).

Treatment with SA significantly up-regulated *PaNPR2* expression at 6 h (2.97-fold) when compared to control samples, returning to baseline levels at 12 h (**Figure 3B**). Plants treated with MeJA showed a progressive decline in the expression of *PaNPR2* with a significant down-regulation at 24 h (0.53-fold) that remained low at 96 h (**Figure 3B**). Similarly, *PaNPR2* was significantly down-regulated at 96 h after infection with *P. cinnamomi* (0.47-fold; **Figure 3B**). Unfortunately the presence of *PaNPR3* could not be reliably detected in either treatment due to low transcript abundance and was therefore omitted from this part of the study.

Significant up-regulation of *PaNPR4* was observed at 6 h (4.63-fold) and 12 h (6.13-fold) with a sharp drop to baseline levels at 18 h (**Figure 3C**). Treatment with MeJA yielded no significant changes in the expression of *PaNPR4*, yet expression seemed to be slightly lower than that of the controls, especially at 96 h (0.44-fold; **Figure 3C**). A significant down-regulation of *PaNPR4* was observed at 18 h *P. cinnamomi* infection (0.53-fold) that remained low until the 96 h time point (**Figure 3C**). Conversely, *PaNPR5* was not significantly altered by any of the hormone treatments, yet was significantly down-regulated by *P. cinnamomi* at 96 h (0.39-fold; **Figure 3D**).

Lastly, expression of *PaPR1* was significantly up-regulated at 6 h (2.71-fold) and 18 h (3.35-fold), with an unexpected slump at 12 h (1.58-fold; **Figure 3E**). This decrease in expression coincided with the highest expression of *PaNPR4* at 12 h and was relieved at 18 h when *PaNPR4* expression returned to basal levels. The expression of *PaPR1* was significantly down-regulated by MeJA at 24 h (0.25-fold) and remained low at 96 h (**Figure 3E**). A significant increase in *PaPR1* expression was seen at 18 h (2.97-fold) followed by a significant decrease at 24 h (0.24-fold) following *P. cinnamomi* inoculation (**Figure 3E**). It was interesting to note that following inoculation with *P. cinnamomi*, the expression of *PaPR1* is similar to that of SA at early time-points and MeJA at later time-points (**Figure 3E**).

### *PaNPR1*-Like Expression in Various Avocado Tissues

*AtBOP1* and *AtBOP2* have been shown to be involved in the growth and development of lateral organs and to accumulate extensively in the proximal parts of these tissues (Hepworth et al., 2005). Thus determining whether any of the *PaNPR1*-like genes are overrepresented in specific tissues could assist in identifying possible *AtNPR1* orthologs by eliminating possible ortholog of the *Arabidopsis BOP* genes in avocado. Consequently, feeder roots, mature stems, mature leaves, unripe fruit as well as stems and leaves from flush growth of mature avocado trees were sampled and basal expression of all five *PaNPR1*-like genes was determined.

**FIGURE 4:**
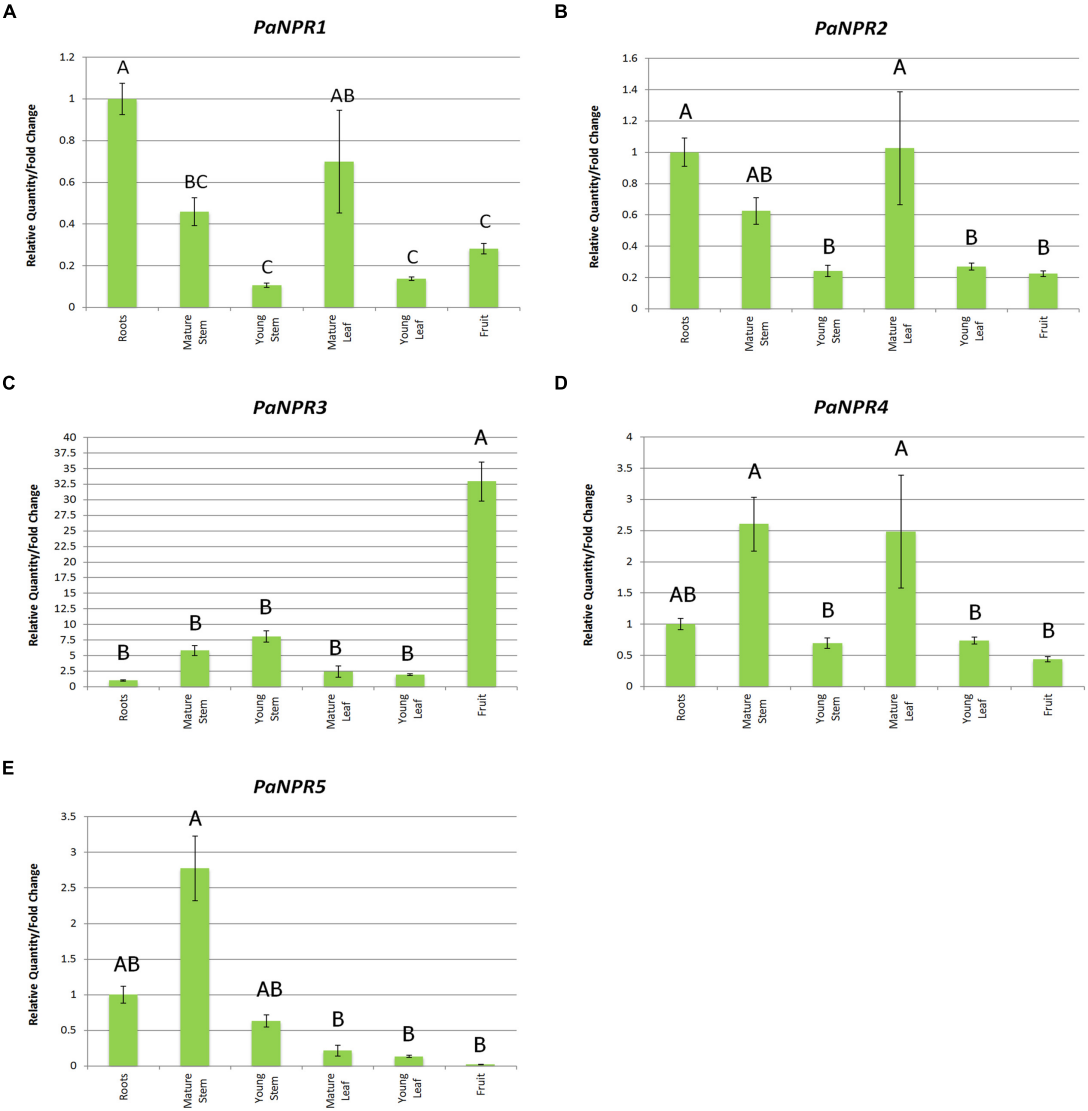
**Expression of *PaNPR1*-like genes in various tissues**. Normalized relative quantities (fold change) for **(A)** *PaNPR1*, **(B)** *PaNPR2*, **(C)** *PaNPR3*, **(D)** *PaNPR4*, and **(E)** *PaNPR5* were calculated using the method described by Pfaﬄ (2001). The response is indicated by vertical bars across six tissues; feeder roots, mature green stems, mature green leaves, unripe fruit as well as stems and leaves from flush growth (young material), and is labeled on the horizontal axis. The expression in all tissues was calibrated using expression in the roots. SE for each bar is shown. Bars represented with the same letter are not significantly different at P < 0.05.

The expression of *PaNPR1, PaNPR2,* and *PaNPR4* was constitutive in all tissues yet higher basal levels were seen in more mature tissues than in younger tissues. The expression of *PaNPR1* was highest in the roots and mature leaves, with the significantly less transcript detected in the young leaves (0.14-fold), young stems (0.11-fold) and unripe fruit (0.28-fold; **Figure 4A**). Similarly, *PaNPR2* had significantly higher expression in roots and mature leaves when compared to young leaves (0.27-fold), young stems (0.24-fold) and unripe fruit (0.22-fold; **Figure 4B**). Expression of *PaNPR4* was significantly higher in mature leaves (2.48-fold) and mature stems (2.61-fold), relative to the young leaves (0.74-fold), young stems (0.70-fold), and unripe fruit (0.44-fold; **Figure 4D**).

Conversely, *PaNPR3* and *PaNPR5* displayed patterns of expression unlike those of the aforementioned *PaNPR1*-like transcripts. Extremely high *PaNPR3* transcript levels were found in the unripe fruit (32.94-fold) relative to the roots, mature stems (5.84-fold), young stems (8.08-fold) and young leaves (1.93-fold; **Figure 4C**). Inversely, expression of *PaNPR5* was the lowest in unripe fruit (0.02-fold) and significantly higher in mature stems (2.77-fold; **Figure 4E**). The expression of this gene was also significantly less in mature leaves (0.21-fold) and young leaves (0.13-fold) when compared to mature stems (**Figure 4E**).

### *PaNPR1*-Like Expression in Tolerant and Susceptible Avocado Rootstocks

The expression of *NPR1*-like genes has been shown to differ significantly between susceptible and tolerant banana cultivars challenged with *Fusarium oxysporum* Schlecht f. sp. *cubense* (Smith) Snyd (*Foc*). Thus determining whether such differences exist between tolerant and susceptible avocado rootstocks could provide insights into molecular differences which may affect *P. cinnamomi* tolerance. Consequently, RNA from both tolerant (Dusa^®^) and susceptible (R0.12) avocado rootstocks, infected with *P. cinnamomi* and harvested at 0 h (uninfected control), 6, 12, and 24 h, was obtained from Engelbrecht et al. (2013).

**FIGURE 5:**
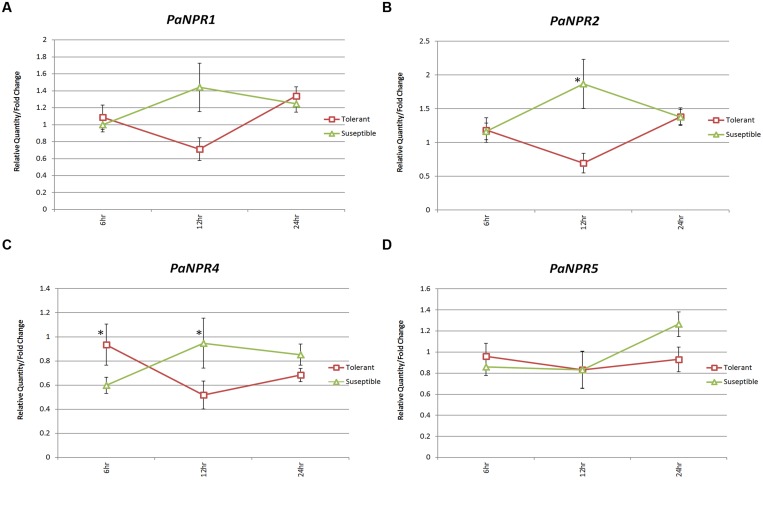
**Expression of *PaNPR1*-like genes in tolerant (Dusa^®^) and susceptible (R0.12) avocado rootstocks infected with *P. cinnamomi***. Normalized relative quantities (fold change) for **(A)** *PaNPR1*, **(B)** *PaNPR2*, **(C)** *PaNPR4,* and **(D)** *PaNPR5* were calculated using the method described by Pfaﬄ (2001). The response is indicated by horizontal lines (red – tolerant, green – susceptible) across three time points; 3, 6, and 24 h, labeled on the horizontal axis. The expression was calibrated using expression at 0 h (uninfected control) which was set to a normalized relative expression of 1. SE for each bar is shown. Significant differences (*P* < 0.05) between control and treated samples is denoted by an asterisk (*) above the data point.

The expression of *PaNPR2* was significantly lower in Dusa^®^ (0.70-fold) as compared to R0.12 (1.87-fold) at 12 h (**Figure 5B**). *PaNPR4* was expressed significantly lower in R0.12 (0.60-fold) when compared to Dusa^®^ (0.94-fold) at 6 h (**Figure 5C**). However, *PaNPR4* was expressed significantly lower in Dusa^®^ (0.52-fold) when compared to R0.12 (0.95) at 12 h (**Figure 5C**). Expression of *PaNPR1* and *PaNPR5* was not significantly different when comparing Dusa and R0.12 at any of the time points (**Figures 5A,D**).

## Discussion

We identified five *NPR1*-like genes in avocado in an attempt to better understand avocado defense response signaling in general, and with regard to the response to PRR. Studies in numerous plant species have highlighted the fundamental involvement of NPR1 in many defense signaling pathways (Cao et al., 1997; Spoel et al., 2003; Le Henanff et al., 2011). This study is the first investigation of the *NPR1*-like gene family in *P. americana*, and sets the foundation for further functional characterization of the NPR1-like protein family in avocado.

The avocado genome contains five identifiable *NPR1*-like genes; these sequences share similar gene structures and protein sequence identities as well as conserved domains and motifs present in *Arabidopsis NPR1*-like sequences. The *PaNPR1*-like gene sequences have similar exon/intron structures compared to the members of the *Arabidopsis NPR1*-like gene family to which each is most similar. Specifically, the exon/intron structures of *PaNPR1*, *PaNPR2,* and *PaNPR4* are comparable to that of *AtNPR1-4*. Interestingly, *PaNPR1* contains an extra, short, exon (exon 3) which could either be an assembly error or a unique aspect of this gene. Furthermore, the exon/intron structures of *PaNPR3* and *PaNPR5* are similar to that of *AtBOP2*. The predicted coding sequences for all five *PaNPR1*-like genes harbor the BTB/POZ and ankyrin repeat domains, characteristic of NPR1-like proteins. These domains are crucial components of NPR1 and provide functions relating to NPR1-dependant co-activation of TGA transcription factors and protein–protein binding (Cao et al., 1997; Rochon et al., 2006). Only PaNPR2 contains the complete IκB phosphodegron motif (DSxxxS) found in AtNPR1, while PaNPR1 contains a lysine substitution at the first serine residue. Both serines of this motif are phosphorylated during SA treatment, leading to proteasome mediated turn-over and degradation of NPR1 (Spoel et al., 2009), suggesting that PaNPR2 may be regulated similarly. However, the effect of the serine to lysine substitution in PaNPR1 is unclear, particularly because basic amino acids such as lysine have been shown to undergo phosphorylation (Ciesla et al., 2011). Similar to AtNPR1, NLS1 sequences are also present in PaNPR1, PaNPR2, and PaNPR4 containing five, four, and three of the amino acids known to be essential to nuclear localization of AtNPR1 (Kinkema et al., 2000). Moreover, PaNPR1, PaNPR2, and PaNPR4 contain highly conserved NIMIN1/2 binding regions and LENRV motifs (Maier et al., 2011). Together these data suggest that PaNPR1, PaNPR2, and PaNPR4 are comparable to AtNPR1 and could possibly partake in the perception of SA and regulation of defense responses in avocado. Seemingly distinct, PaNPR3 and PaNPR5 do not contain these motifs and conserved regions, similar to the AtBOP proteins. The substantial difference in protein length and sequence composition of this subset of proteins suggests functionally diverse roles from PaNPR1, PaNPR2, and PaNPR4, and can be suggested that these proteins may be involved in certain aspects of tissue development as seen in AtBOP1 and AtBOP2 (Hepworth et al., 2005).

Phylogenetic analysis reveals that all five PaNPR1-like proteins group with other known NPR1-like sequences, clustering into three distinct clades (Peraza-Echeverria et al., 2012). PaNPR1 and PaNPR2 fall within the same clade as AtNPR1, which is a known positive regulator of SAR (Cao et al., 1997, 1998). PaNPR4 groups with AtNPR3 and AtNPR4 in the second clade. NPR1-like proteins within this group have been shown to negatively regulate SAR (Zhang et al., 2006), yet are able to perceive SA and are vital in mounting SAR (Fu et al., 2012). The last two, PaNPR3 and PaNPR5, fall within the third clade together with AtBOP1 and AtBOP2, which are known for their involvement in development of lateral organs (Hepworth et al., 2005). This phylogenetic analysis provides a second line of evidence suggesting possible functional distinctions between members of the PaNPR1-like protein family.

This study further describes the transcriptional response of the *PaNPR1*-like genes to SA, MeJA, and *P. cinnamomi* treatments as well as their expression levels in different tissues. Surprisingly, *PaNPR1* was down regulated by SA at 12 h, contrasting with *AtNPR1*, which was up-regulated approximately twofold 24 h after SA application in *Arabidopsis* (Cao et al., 1998). This may point to an alternative function of *PaNPR1* during defense responses and warrants further investigation. In banana cultivars which are resistant to *Foc*, NPR1 is up-regulated to a greater extent and at earlier time points after SA treatment than in susceptible cultivars (Endah et al., 2008). Similarly, *PaNPR2* was up-regulated at the earliest time point after SA treatment in the PRR tolerant avocado rootstock Dusa^®^. Additionally, up-regulation of *PaNPR2* corresponded to an increase in *PaPR1* gene expression. Similarly, *PaNPR4* was up-regulated soon after SA application and reached peak expression at 12 h. Interestingly, the highest expression of *PaNPR4* corresponded to a substantial decrease in *PaPR1* expression, suggesting that PaNPR4 may negatively regulate the expression of *PaPR1*. Treatment with MeJA led to decreased transcript abundance for *PaNPR1*, *PaNPR2,* and *PaNPR4* for extended periods of time, opposite to that seen in rice and banana (Yuan et al., 2007; Endah et al., 2008). The regulation of NPR1 during antagonistic cross-talk between SA and JA mediated defense responses may thus differ between some monocot and dicot plants. Interestingly, the regulation of *PaNPR1*, *PaNPR2,* and *PaNPR4* during *P. cinnamomi* treatment had expression patterns similar to that of SA treatment at earlier time points (6–18 h) and JA treatment at later time points (24–96 h). This could indicate the point at which *P. cinnamomi* switches from a biotrophic to a necrotrophic life stage, thus activating the SA and JA pathways, respectively.

Oddly, the induced expression of *PaNPR2* and *PaNPR4* during SA treatment was not observed during infection with *P. cinnamomi*. In our opinion three possible explanations exist: (1) PaNPR2 and PaNPR4 might be predominantly regulated at the protein level, (2) these proteins might not be involved in defense responses against *P. cinnamomi* or, (3) *P. cinnamomi* suppresses expression of these genes in order to promote successful host invasion. It has been noted that NPR1 is subject to extensive post-translational regulation (Mou et al., 2003; Spoel et al., 2009), thus changes in expression might not reflect the factual role of PaNPR1, PaNPR2, or PaNPR4 during defense response. Furthermore, *Phytophthora* species have been known to alter host gene expression in order to suppress host defense pathways and mediate infection (Oßwald et al., 2014).

A common trend in the expression of *PaNPR1*, *PaNPR2,* and *PaNPR4* in various tissues was seen; transcript levels of these genes were significantly higher in mature tissues than immature tissues, an observation that may be explained by the establishment of SAR in mature tissues. Expression of *PaNPR3* was undetectable in the roots in any of the treatments, yet this gene was expressed at much higher levels in aerial tissues, with the highest levels being detected in fruit. On the other hand, while *PaNPR5* was readily detected in the roots, it was unresponsive to SA or MeJA treatments and significantly down-regulated by *P. cinnamomi* during later time points. These data support our initial hypothesis that PaNPR3 and PaNPR5 are unlikely to be involved in defense responses, and are instead more likely to be involved in development of certain tissues.

Finally, significant differences in the expression of *PaNPR2* and *PaNPR4* were observed when comparing tolerant (Dusa^®^) and susceptible (R0.12) avocado rootstock cultivars. The expression of *PaNPR2* and *PaNPR4* is significantly lower at 12 h after infection in Dusa^®^ when compared to R0.12. In our opinion these observations could be explained when considering *P. cinnamomi* switching from a biotrophic to a necrotrophic life cycle. In this case, increased expression of *PaNPR1-*like defense related genes would likely suppress the JA/ET pathway and prevent effective control of *P. cinnamomi*. Thus it is conceivable that *P. cinnamomi* switches to a necrotrophic life cycle somewhere around 12 h after infection and that Dusa^®^ reacts to this change more quickly than R0.12. This would explain, at least to some extent, tolerance in Dusa^®^ and susceptibility in R0.12.

This study provides evidence assisting in the preliminary functional annotation of five newly discovered *NPR1*-like genes from avocado. Sequence structure and homology as well as phylogenetic analyze suggest that three PaNPR1-like proteins may be involved in defense responses, while the remaining two are most likely involved tissue development. Hormone and *P. cinnamomi* treatments, as well as expression in various tissues provide support for this and allow future research to focus on defense related PaNPR1-like proteins. Future efforts would be focused on intracellular interactions and localization of defense related PaNPR1-like proteins as well as the effect of overexpressing defense related *PaNPR1*-like genes in wild-type and *npr1* mutant *Arabidopsis*. Information from this and future studies could aid in understanding PRR tolerance and lead to the development of more tolerant avocado rootstocks.

## Author Contributions

RB drafted the manuscript, performed conceptual, and experimental design as well as performed the experimental work. WM assisted in expression analysis and experimental design. JE performed experimental work. BR provided general supervision and assisted in drafting the manuscript. EL sequenced the genome and provided the *PaNPR1*-like sequences. NvdB conceived the study, experimental design and assisted in drafting the manuscript. All authors contributed to and approved the final manuscript.

## Conflict of Interest Statement

The authors declare that the research was conducted in the absence of any commercial or financial relationships that could be construed as a potential conflict of interest.

## Footnotes

1 http://faostat3.fao.org/faostat-gateway/go/to/home/E

2 http://imed.med.ucm.es/Tools/sias.html

3 http://www.ncbi.nlm.nih.gov/genbank/

4 http://www.ncbi.nlm.nih.gov/

5 http://www.socr.ucla.edu
